# Is It Relevant to Keep Advocating Visual Inspection of the Cervix With Acetic Acid for Primary Cervical Cancer Screening in Limited-Resource Settings?

**DOI:** 10.1200/JGO.17.00048

**Published:** 2017-10-16

**Authors:** Joel Fokom Domgue, Fidel A. Valea

**Affiliations:** **Joel Fokom Domgue**,University Hospital Centre, Yaounde, Cameroon, and National Cancer Institute, Rockville, MD; and; **Fidel A. Valea**, Virginia Tech Carilion School of Medicine, Roanoke, VA.

Although the burden of cervical cancer has significantly declined in high-income settings, this preventable disease has remained a public health priority in most developing countries, where it is still among the commonest and deadliest women’s cancers.^1^ Despite the huge number of resources that have been invested over the past decades, the incidence of cervical cancer has continued to increase in limited-resource settings, and projections for the coming years confirm that this tendency^2^ will remain until high-impact interventions (including vaccination against human papilloma virus [HPV], which is the main factor leading to cervical cancer [referred to as primary prevention]) are widely and effectively implemented. While the introduction of vaccines is relatively recent and is still being investigated (ie, number of doses and interval between doses, target age range and population groups, long-term efficacy, and adverse effects), screening and proper management of precancerous lesions (referred to as secondary prevention) have been extensively assessed and promoted around the world to curb this problem.^1^

Given the difficulties to establish and sustain cytology-based screening programs that have achieved significant results in developed settings, alternative methods of cervical screening that could better meet the needs of high-burden but resource-constrained areas have been developed and largely studied.^3-5^ Considered so far to be one of the most appropriate methods for a screen-and-treat approach (a concept widely endorsed as a secondary prevention strategy suited for limited-resource settings, which links screening with management of precancerous lesions to reduce the loss to follow-up associated with multivisit schemes), visual inspection of the cervix with acetic acid (VIA) has been widely promoted and recommended for many decades as a viable alternative to cytology in resource-constrained countries.^6,7^ Advantages of VIA include its relative simplicity (it can be performed by midlevel health care workers) and low cost (it requires short training and modest equipment), as well as the possibility of providing results immediately and thus offering treatment to patients during the same visit.^7,8^

This enthusiasm about VIA has been reinforced by the results of many demonstration projects in high-burden settings, most of which have shown that the performance of this screening method was on average higher than that of cytology,^7,9^ and that the method was overall well accepted and feasible in limited-resource settings.^10,11^ Thus, significant investments have been made by private and public donors, national and international governmental or nongovernmental organizations, and the scientific community to promote this so-called promising method of screening, to such an extent that a growing number of high-burden countries have endorsed and sponsored a single-visit screening approach, with VIA linked to treatment of cervical precancerous lesions, in their national cervical cancer control programs.^7^

As these programs have been implemented and expanded, many issues that had not been sufficiently taken into account at the outset or whose impact on the effectiveness of the strategy had been initially minimized in pilot projects, emerged when the VIA-based see-and-treat strategy was used in the actual conditions of health systems in these countries.^12^ These problems highlighted the limitations associated with a VIA-based strategy, and questioned both its sustainability and effectiveness in reducing the burden of cervical cancer.

From a screening method or an epidemiologic perspective, the limitations of this strategy include the subjective nature of VIA, which leads to high variability in the performance of providers, frequent false-positive results, suboptimal sensitivity and specificity, overtreatment, and poor performance in postmenopausal women.^1,7^ In addition, the absence of validated quality assurance methods hampers standardization of VIA and assessment of providers’ abilities to accurately perform this test. From a programmatic or implementation perspective, the feasibility of scaling up a VIA-based see-and-treat approach was increasingly challenged in the field, because it does not dispense providers with the important step of counseling or careful explanation about the need for follow-up (especially in case of treatment) and the risk of adverse events associated with treatment procedures.^1,12^ From a prognostic or health impact perspective, although an early trial has shown that VIA may have a positive impact on reducing mortality and morbidity related to cervical cancer,^13^ the authors acknowledged that these results were only achieved through rigorous study procedures that were put in place. These study features that are part of clinical trials (ie, VIA practiced by well-trained health care workers with regular refreshing and supervision, short waiting time for participants, high rate of acceptance of screening and compliance with treatment, low rates of adverse effects and patients lost to follow-up, and gratuity of procedures) are actually difficult to maintain in real practice. Moreover, recent clinical trials have found more conflicting results on the potential of a VIA-based strategy to have an effect on the burden of this preventable disease.^14,15^ Despite this growing body of evidence, several organizations (including the WHO and scholarly societies) continue to advocate the use of VIA for cervical screening in limited-resource settings.^16^ If this recommendation could be understandable some years ago, when alternative solutions to VIA were out of scope, recent scientific and technological advances have allowed the development of new screening tools that are increasingly available and affordable.

Indeed, molecular screening based on the detection of high-risk HPV in exfoliated cervico-vaginal cells (referred to as HPV testing) has made a lot of progress and is nowadays considered the most accurate screening method for cervical cancer. HPV tests rely on more automated procedures with less human involvement and are more sensitive and reproducible than VIA.^17,18^ Thus, HPV testing provides greater reassurance to women that they are not at substantial risk of cervical cancer, which allows larger intervals between testing and consequently reduces the lifetime number of required screening rounds. This is an important consideration for limited-resource settings because a small number of lifetime screening rounds might improve the practicability of the strategy in areas with an insufficient workforce, while reducing the cost of the program.^19,20^ In addition, HPV testing can be performed in postmenopausal women without significant loss of accuracy, and the specificity of HPV testing is improved when it is done from 30 years of age.^7,18^ Another major advantage of HPV testing is the possibility of self-sampling, which is an additional important consideration in limited-resources settings because it can substantially increase the participation and adherence of underserved and hard-to-reach populations to screening.^21,22^

While HPV tests were initially expensive and required sophisticated laboratory infrastructures and highly skilled personnel to be performed, recent advancements in the field of molecular biology and genetic engineering have allowed the development of more simplified and affordable HPV assays. Some of these assays have been designed for use in resource-constrained settings and can be used at the point of care because they can produce results within a short period of time (1 to 3 hours) and do not require extensive human expertise or technical equipment.^23-25^ The value of point-of-care testing is to avoid the costs and attrition that occurs when women must be recalled to get their results. Although improvements in the development of simpler and faster HPV tests are expected in future years, the most recent models available may allow the incorporation of HPV testing into screening programs in resource-constrained countries.

Indeed, there are now validated HPV assays that are inexpensive enough to compete with VIA for use as primary screening tools in limited-resource settings. As an example, in an ongoing cervical screening program using VIA in West Africa, the fees charged to women for VIA vary between 3 and 8 US dollars.^26^ Meanwhile, the price of an HPV testing procedure in ongoing screening programs in West Africa and Latin America is between 5 and 6 US dollars per woman.^25^ Moreover, companies that make HPV assays might be willing to offer more affordable prices when HPV-based screening strategies are implemented nationwide as part of programs wherein a high number of women are screened. With respect to staff training, experts established that a training shorter than 1 week including didactic lectures, practical sessions, and hands-on training was sufficient to train personnel to independently and properly perform HPV testing procedures,^27^ compared with a training period of at least 2 weeks for VIA.^28^ In addition, periodic refreshing may be more demanding for VIA than for point-of-care HPV testing, because the latter is an essentially automated procedure. Concerning the reliability of HPV testing in rural settings, some HPV assays have been designed for use in limited-resource settings where the electricity supply is suboptimal and a cold chain is poorly available.^23,24^

Recently, ASCO published its new resource-stratified clinical practice guidelines for secondary prevention of cervical cancer.^29,30^ This is a practical and useful adaptation of the WHO’s guidelines for cervical cancer screening; it is based on the four-resource tier approach developed by the Breast Health Global Initiative.^31^ Interestingly, ASCO guidelines provide guidance on the number of lifetime screenings, the age groups targeted for screening, and the management options, in relation to the level of resources available. This is a major advance in helping decision makers choose an appropriate prevention strategy, especially in limited-resource settings.

While we agree with the recommendation that HPV testing should be the preferred approach for cervical cancer screening, the argument that VIA in the basic-resource settings can be used to build health care infrastructures until HPV testing becomes available seems questionable. To support this recommendation, the authors refer to the results of a large trial that demonstrated the positive effect of VIA screening on cervical cancer mortality, but not on its incidence.^14^ However, they did not seem to take into account the results of another large trial that reported no significant reduction in the numbers of cases or deaths from cervical cancer after a single round of VIA or cytology.^15^ Therefore, the reduction in cervical cancer mortality observed in the trial by Shastri et al^14^ could be explained, at least in part, by the high number of VIA-based screening rounds (four in total) and the short interval between two consecutive screening rounds (24 months on average), which is hardly achievable in the basic-resource settings. Thus, when recommending one to three screenings per lifetime in the basic-resource settings, the authors of these guidelines should recognize that the use of a VIA-based strategy under these circumstances has no proven benefit in terms of mortality from or incidence of cervical cancer. Accordingly, it seems irrelevant to keep supporting the use of VIA for cervical cancer screening with the hope that it might improve health care infrastructures in the basic-resource settings. Indeed, this would mean advising decision makers to invest scarce resources in building a health system using a strategy with uncertain health implications for the population, while better options, such as lower-cost and point-of-care HPV assays that have a proven effect on population health, are readily available. This does not seem to be the best recommendation for limited-resource settings, particularly because cost-effectiveness studies have demonstrated the advantage in terms of health benefits of an HPV-based screening strategy that yields high population coverage over a VIA-based screening strategy.^32,33^

Supposedly, even if decision makers approved this approach, its applicability on the ground would raise other concerns. From the population perspective, if women are told that the VIA strategy has uncertain health benefits but is rather intended to help build health care infrastructures to prepare for future incorporation of more effective screening methods, in addition to the ethical issues that this may pose, their willingness to participate in the program might be significantly lowered. This could be compared with a vaccination strategy where the government decides to administer distilled water to the target population, explaining that the distilled water might not be beneficial in terms of preventing a disease, but that it can help build a vaccine delivery system that will be operational when effective vaccines become available.

In conclusion, we would rather suggest, in the basic-resource settings, to consider introducing an HPV-based screening strategy linked to treatment of lesions, with the most affordable HPV assays that have been validated for cervical cancer screening,^23-25^ and to build the health care infrastructures that better match the needs of an HPV-based strategy. In settings where no screening program exists, the introduction of HPV testing should be considered at the same level of priority as building the health care infrastructure. In settings where a VIA-based program is already implemented, HPV testing should be introduced for screening and VIA used to manage women whose screens were positive. If we want to gain time and save more lives in the fight against this disease, our prevention policies should be more ambitious and updated in light of the most recent scientific and technological breakthroughs.

## References

[1] Denny L de Sanjose S Mutebi M et al Interventions to close the divide for women with breast and cervical cancer between low-income and middle-income countries and high-income countries Lancet 389 861 870 2017 27814963 10.1016/S0140-6736(16)31795-0

[2] Global Burden of Disease Cancer Collaboration Fitzmaurice C Allen C et al Global, regional, and national cancer incidence, mortality, years of life lost, years lived with disability, and disability-adjusted life-years for 32 cancer groups, 1990 to 2015: A systematic analysis for the Global Burden of Disease Study JAMA Oncol 3 524 548 2017 27918777 10.1001/jamaoncol.2016.5688 PMC6103527

[3] Gelband H Jha P Sankaranarayanan R et al Screening for cancer: Considerations for low and middle-income countries Cancer: Disease Control Priorities Vol. 3Chapter 12. The International Bank for Reconstruction and Development/The World Bank Washington, DC 2015 211 222 26913335

[4] Adefuye PO Broutet NJ de Sanjosé S et al Trials and projects on cervical cancer and human papillomavirus prevention in sub-Saharan Africa Vaccine 31 Suppl 5 F53 F59 2013 24331748 10.1016/j.vaccine.2012.06.070

[5] Cuzick J Arbyn M Sankaranarayanan R et al Overview of human papillomavirus-based and other novel options for cervical cancer screening in developed and developing countries Vaccine 26 Suppl 10 K29 K41 2008 18847555 10.1016/j.vaccine.2008.06.019

[6] Sankaranarayanan R Nessa A Esmy PO et al Visual inspection methods for cervical cancer prevention Best Pract Res Clin Obstet Gynaecol 26 221 232 2012 22075441 10.1016/j.bpobgyn.2011.08.003

[7] Sherris J Wittet S Kleine A et al Evidence-based, alternative cervical cancer screening approaches in low-resource settings Int Perspect Sex Reprod Health 35 147 154 2009 19805020 10.1363/ifpp.35.147.09

[8] World Health Organization Comprehensive Cervical Cancer Control: A Guide to Essential Practice WHO Geneva 2006 25642554

[9] Fokom-Domgue J Combescure C Fokom-Defo V et al Performance of alternative strategies for primary cervical cancer screening in sub-Saharan Africa: Systematic review and meta-analysis of diagnostic test accuracy studies BMJ 351 h3084 2015 26142020 10.1136/bmj.h3084 PMC4490835

[10] Gaffikin L Blumenthal PD Emerson M et al Safety, acceptability, and feasibility of a single-visit approach to cervical-cancer prevention in rural Thailand: A demonstration project Lancet 361 814 820 2003 12642047 10.1016/s0140-6736(03)12707-9

[11] Sankaranarayanan R Rajkumar R Esmy PO et al Effectiveness, safety and acceptability of ‘see and treat’ with cryotherapy by nurses in a cervical screening study in India Br J Cancer 96 738 743 2007 17311015 10.1038/sj.bjc.6603633 PMC2360066

[12] Fokom-Domgue J Vassilakos P Petignat P Is screen-and-treat approach suited for screening and management of precancerous cervical lesions in Sub-Saharan Africa? Prev Med 65 138 140 2014 24879892 10.1016/j.ypmed.2014.05.014

[13] Sankaranarayanan R Esmy PO Rajkumar R et al Effect of visual screening on cervical cancer incidence and mortality in Tamil Nadu, India: A cluster-randomised trial Lancet 370 398 406 2007 17679017 10.1016/S0140-6736(07)61195-7

[14] Shastri SS Mittra I Mishra GA et al Effect of VIA screening by primary health workers: Randomized controlled study in Mumbai, India J Natl Cancer Inst 106 dju009 2014 24563518 10.1093/jnci/dju009 PMC3982783

[15] Sankaranarayanan R Nene BM Shastri SS et al HPV screening for cervical cancer in rural India N Engl J Med 360 1385 1394 2009 19339719 10.1056/NEJMoa0808516

[16] Santesso N Mustafa RA Schünemann HJ et al World Health Organization Guidelines for treatment of cervical intraepithelial neoplasia 2-3 and screen-and-treat strategies to prevent cervical cancer Int J Gynaecol Obstet 132 252 258 2016 26868062 10.1016/j.ijgo.2015.07.038

[17] Tota JE Bentley J Blake J et al Introduction of molecular HPV testing as the primary technology in cervical cancer screening: Acting on evidence to change the current paradigm Prev Med 98 5 14 2017 28279264 10.1016/j.ypmed.2016.11.029

[18] Arbyn M Ronco G Anttila A et al Evidence regarding human papillomavirus testing in secondary prevention of cervical cancer Vaccine 30 Suppl 5 F88 F99 2012 23199969 10.1016/j.vaccine.2012.06.095

[19] Kuhn L Denny L The time is now to implement HPV testing for primary screening in low resource settings Prev Med 98 42 44 2017 28279263 10.1016/j.ypmed.2016.12.030 PMC5578476

[20] Cuzick J Clavel C Petry KU et al Overview of the European and North American studies on HPV testing in primary cervical cancer screening Int J Cancer 119 1095 1101 2006 16586444 10.1002/ijc.21955

[21] Zhao FH Lewkowitz AK Chen F et al Pooled analysis of a self-sampling HPV DNA Test as a cervical cancer primary screening method J Natl Cancer Inst 104 178 188 2012 22271765 10.1093/jnci/djr532 PMC3274511

[22] Arrossi S Thouyaret L Herrero R et al Effect of self-collection of HPV DNA offered by community health workers at home visits on uptake of screening for cervical cancer (the EMA study): A population-based cluster-randomised trial Lancet Glob Health 3 e85 e94 2015 25617202 10.1016/S2214-109X(14)70354-7

[23] Lorenzi AT Fregnani JH Possati-Resende JC et al Can the careHPV test performed in mobile units replace cytology for screening in rural and remote areas? Cancer Cytopathol 124 581 588 2016 27070446 10.1002/cncy.21718

[24] Toliman P Badman SG Gabuzzi J et al Field evaluation of Xpert HPV point-of-care test for detection of human papillomavirus infection by use of self-collected vaginal and clinician-collected cervical specimens J Clin Microbiol 54 1734 1737 2016 27076663 10.1128/JCM.00529-16 PMC4922075

[25] Fokom Domgue J Schiffman M Wentzensen NH et al Assessment of a new lower-cost real-time PCR assay for detection of high-risk human papillomavirus: Useful for cervical screening in limited-resource settings? J Clin Microbiol 55 2348 2355 2017 28515214 10.1128/JCM.00492-17 PMC5527412

[26] DeGregorio G Manga S Kiyang E et al Implementing a fee-for-service cervical cancer screening and treatment program in Cameroon: Challenges and opportunities Oncologist 22 850 859 2017 28536303 10.1634/theoncologist.2016-0383 PMC5507645

[27] Gage JC Ajenifuja KO Wentzensen N et al Effectiveness of a simple rapid human papillomavirus DNA test in rural Nigeria Int J Cancer 131 2903 2909 2012 22473652 10.1002/ijc.27563 PMC3404249

[28] Poli UR Bidinger PD Gowrishankar S Visual inspection with acetic acid (VIA) screening program: 7 years experience in early detection of cervical cancer and pre-cancers in rural South India Indian J Community Med 40 203 207 2015 26170547 10.4103/0970-0218.158873 PMC4478664

[29] Castle PE Jeronimo J Temin S et al Screening to prevent invasive cervical cancer: ASCO resource-stratified clinical practice guideline J Clin Oncol 35 1250 1252 2017 28135111 10.1200/JCO.2016.71.6563

[30] Jeronimo J Castle PE Temin S et al Secondary prevention of cervical cancer: American Society of Clinical Oncology resource-stratified clinical practice guideline summary J Oncol Pract 13 129 133 2017 27845871 10.1200/JOP.2016.017889

[31] Anderson BO Shyyan R Eniu A et al Breast cancer in limited-resource countries: An overview of the Breast Health Global Initiative 2005 guidelines Breast J 12 Suppl 1 S3 S15 2006 10.1111/j.1075-122X.2006.00199.x 16430397

[32] Campos NG Castle PE Wright TC Jr et al Cervical cancer screening in low-resource settings: A cost-effectiveness framework for valuing tradeoffs between test performance and program coverage Int J Cancer 137 2208 2219 2015 25943074 10.1002/ijc.29594 PMC4910518

[33] Mezei AK Armstrong HL Pedersen HN et al Cost-effectiveness of cervical cancer screening methods in low- and middle-income countries: A systematic review Int J Cancer 141 437 446 2017 28297074 10.1002/ijc.30695

